# Evolution of the Clinical Profile and Outcomes of Unvaccinated Patients Affected by Critical COVID-19 Pneumonia from the Pre-Vaccination to the Post-Vaccination Waves in Italy

**DOI:** 10.3390/pathogens11070793

**Published:** 2022-07-14

**Authors:** Cecilia Calabrese, Anna Annunziata, Domenica Francesca Mariniello, Antonietta Coppola, Angela Irene Mirizzi, Francesca Simioli, Corrado Pelaia, Lidia Atripaldi, Gaia Pugliese, Salvatore Guarino, Giuseppe Fiorentino

**Affiliations:** 1 Department of Translational Medical Sciences, University of Campania ‘Luigi Vanvitelli’, A.O.R.N. Ospedali dei Colli, 80131 Napoli, Italy; nikamariniello93@gmail.com (D.F.M.); lidia.atripaldi@virgilio.it (L.A.); pugliese.gaia@gmail.com (G.P.); 2 Department of Intensive Care, A.O.R.N. Ospedali dei Colli, 80131 Napoli, Italy; anna.annunziata@gmail.com (A.A.); antonietta.coppola84@gmail.com (A.C.); angela.mirizzi1989@gmail.com (A.I.M.); francesimioli@gmail.com (F.S.); giuseppefiorentino1@gmail.com (G.F.); 3 Respiratory Medicine Unit, University “Magna Græcia” of Catanzaro, 88100 Catanzaro, Italy; pelaia.corrado@gmail.com; 4 Department of Radiology, Monaldi Hospital, A.O.R.N. Ospedali dei Colli, 80131 Napoli, Italy; sag1981@libero.it

**Keywords:** COVID-19 pandemic, SARS-CoV-2 variants, SARS-CoV-2 vaccination, COVID-19 pneumonia, clinical characteristics, outcomes, respiratory failure, HRCT score

## Abstract

The vaccination campaign and the new SARS-CoV-2 variants may have changed the clinical profile and outcomes of patients admitted to sub-intensive unit care. We conducted a retrospective study aimed to compare the clinical and radiological features of unvaccinated critical COVID-19 patients hospitalized during the last pandemic wave (December 2021–February 2022, No-Vax group) and before starting the vaccination campaign (March–December 2020, Pre-Vax group). The No-Vax group was also compared with vaccinated patients of the same pandemic wave (Vax group). With respect to the Pre-Vax group, the No-Vax group contained a higher percentage of smokers (*p* = 0.0007) and a lower prevalence of males (*p* = 0.0003). At admission, the No-Vax patients showed both a higher CT score of pneumonia and a worse severe respiratory failure (*p* < 0.0001). In the No-Vax group, a higher percentage of deaths occurred, though this was not significant. In comparison with the No-Vax group, the Vax patients were older (*p* = 0.0097), with a higher Charlson comorbidity index (*p* < 0.0001) and a significantly lower HRCT score (*p* = 0.0015). The percentage of deaths was not different between the two groups. The No-Vax patients showed a more severe disease in comparison with the Pre-Vax patients, and were younger and had fewer comorbidities than the Vax patients.

## 1. Introduction

The novel SARS-CoV-2 infection reported in Wuhan, China, in December 2019 and has spread rapidly around the world during the last two years [1]. Italy was one the first countries to be involved by the COVID-19 pandemic and, after the first cases were identified in February 2020, several waves of pandemics occurred; we are actually experiencing the fourth wave.

Since December 2020, when the first variant of concern, known as Alpha (B.1.1.7), was identified, other variants have been recognized [2]. The latest two, Delta (B.1.617.2) and Omicron (B.1.1.529), seem to be more transmissible than the earlier; Delta has been associated with decreased levels of morbidity and mortality in Europe and the USA, but the impact of the new Omicron variant, designated by WHO on 26 November 2021, is under investigation [3].

On 27 December 2020, a coordinated vaccination campaign started in the European Union countries. It initially prioritized high-risk individuals, such as elderly subjects and patients with several chronic comorbidities, then progressively involved younger and healthier subjects [4,5]. In February 2022, when more than 11,000,000 COVID-19 cases had been identified in Italy with approximately 149,000 deaths, about 88% of the Italian population over the age of 12 had since completed the vaccination cycle and 83% had received the booster dose [6,7].

Several studies have demonstrated the efficacy of anti-SARS-CoV-2 vaccines in preventing infection, hospitalization, and the need for emergency care [8]. In a recent multicenter study performed in the USA between December 2020 and April 2021 involving 11,834 COVID-19 patients, a significantly higher proportion of unvaccinated patients (91.9%) required emergency care and/or hospitalization compared to fully vaccinated patients with a breakthrough SARS-CoV-2 infection (1.1%); however, once hospitalized, vaccination status did not appear to reduce the need for intensive care unit admission or mechanical ventilation, or reduce hospital death [9].

Studies performed at the beginning of the pandemic have identified advanced age, male gender, and comorbidities, such as arterial hypertension, as the main risk factors associated to the most severe forms of COVID-19 pneumonia [10]. However, it is not known whether these risk factors have changed considering that the older, more fragile and comorbid patients should be vaccinated, and new viral variants are circulating. For example, a previous study demonstrated a shift in the age distribution of COVID-19 deaths induced by the vaccination campaign in countries that prioritized the elderly, with increased rates of deaths among subjects 0–69 years old in comparison with those older than 70 years [11]. Thus, the main aim of the present study was to compare the clinical features, the disease course, and the outcomes of critical COVID-19 pneumonia occurring in unvaccinated patients during the last pandemic wave and before the start of the vaccination campaign. Furthermore, to evaluate the effects of anti-SARS-CoV-2 vaccines, we compared unvaccinated and vaccinated patients hospitalized during the fourth, most recent COVID-19 wave.

## 2. Materials and Methods

We conducted a cross-sectional single-center retrospective study involving critical COVID-19 patients admitted to the Sub-Intensive Care Unit of A.O.R.N. Ospedali dei Colli, Cotugno Hospital, Naples, Italy, during the first COVID-19 pandemic waves before the start of the vaccination campaign (from March 2020 to December 2020)—named the Pre-Vax group—and during the fourth, most recent wave (between December 2021 and February 2022)—designated, according to the vaccination status, as the unvaccinated (No-Vax) and vaccinated (Vax) groups.

The study enrolled adult patients affected by critical COVID 19 pneumonia as shown by a confirmed diagnosis of SARS-CoV-2 infection via real-time reverse transcription-polymerase chain reaction (RT-PCR) testing on nasopharyngeal swabs, an evidence of interstitial pneumonia on high-resolution computed tomography (HRCT) of the chest and a respiratory failure with a ratio between the partial pressure of arterial oxygen and the fraction of inspired oxygen(PaO_2_/FiO_2_) ≤ 300 mmHg [12].

All patients received the best of care according to the recommendations of the guidelines progressively updated during the COVID-19 pandemic [12,13]. In particular, as reported in our previous study, patients enrolled in the Pre-Vax group were also treated with systemic corticosteroid therapy as well as those of the No-Vax and Vax groups, but, in contrast, the Pre-Vax group did not receive a prophylactic dose of anticoagulants [14].

At hospital admission, the following data of the entire study group were collected from medical records on an electronic database:

1. Demographic characteristics (age, sex, and BMI);
2. Smoking status (actual and former or never smokers);
3. Comorbidities as arterial hypertension, obesity, diabetes, bronchial asthma, chronic obstructive pulmonary disease (COPD), malignancies, and others. The Charlson comorbidity index (CCI) was calculated by summing the assigned weighted score of 19 comorbid conditions: higher scores indicated a more severe condition and consequently a worse ten-year survival [15];
4. Chest HRCT score according to Chung et al.: the total severity score, ranging from 0 to 20,was calculated by adding the score of each of the five lung lobes, as follows: score zero (no lobe involvement), score one (minimal involvement, 1–25%), score two (mild involvement, 26–50%), score three (moderate involvement, 51–75%), score four (severe involvement, 76–100%) [16];
5. PaO_2_/FiO_2_ ratio and respiratory supports: non-invasive ventilation (NIV), continuous positive airway pressure (cPAP), high flow nasal cannula (HFNC), Venturi mask, or nasal cannula;
6. Laboratory data: white blood cells (WBC) with neutrophil and lymphocyte percentages and neutrophil–lymphocyte ratio (NLR), C-reactive protein (CRP), interleukin (IL)-6, dimer D, procalcitonin, aspartate aminotransferase (AST), alanine aminotransferase (ALT), lactate dehydrogenase (LDH), nitrogen urea, creatinine, and glycemia;
7. Days between the first positive SARS-CoV-2 RT-PCR test and the hospital admission and days of hospital stay;
8. The occurrence of pulmonary embolism during the hospitalization;
9. The exitus (death or survival).

In the Vax group, the vaccination-related features (type of vaccine, date of vaccination, number of doses) were recorded.

In the No-Vax and Vax groups, the SARS-CoV-2 viral variants were identified.

### Statistical Analysis

Data were expressed as mean ± standard deviation (SD) if normally distributed, otherwise as median values with interquartile ranges (IQR). The Anderson–Darling test was applied to investigate if data were normally distributed. Dunnett’s multiple comparison test and the Friedman test were used to compare variables, when appropriate. The χ^2^ test was performed for nominal data. A *p* value of lower than 0.05 was considered to be statistically significant. Statistical analysis was performed using Prism Version 9.3.1 (GraphPad Software Inc., San Diego, CA, USA).

## 3. Results

A total of 277 critical COVID-19 patients were enrolled: 132 in the Pre-Vax group, 105 in the No-Vax group, and 40 in the -Vax group. In the Vax group, 6 patients received one dose, 29 two doses, and 5 three doses of anti-SARS-CoV-2 vaccines, and all patients received a vaccine dose >14 days before a positive test.

All results are shown in Table 1.

### 3.1. No-Vax vs. Pre-Vax Group

**Figure 1:**
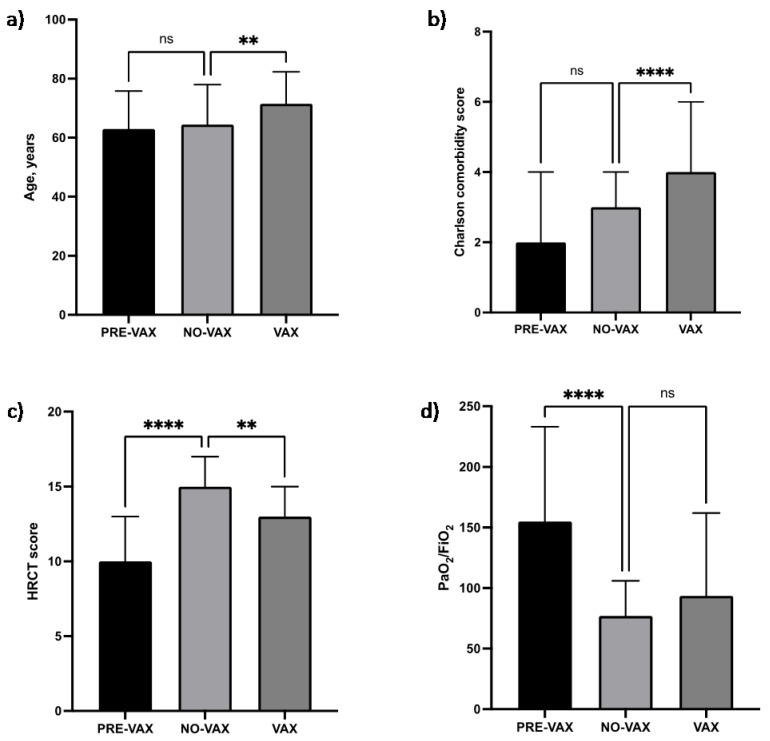
(**a**) The age of patients was significantly higher in the Vax group compared to the No-Vax group. (**b**) The Charlson comorbidity index was significantly higher in the Vax group compared to the No-Vax group. (**c**) The HRCT score was significantly higher in the No-Vax group in comparison with both the Pre-Vax and Vax groups. (**d**) The PaO_2_/FiO_2_ratio was significantly higher in the Pre-Vax group compared to the No-Vax group. ** *p* < 0.01; **** *p* < 0.0001; ns: not significant. Abbreviations: HRCT, high-resolution computed tomography; PaO_2_/FiO_2_, partial pressure of oxygen in the arterial blood/fraction of inspired oxygen.

By comparing the No-Vax with the Pre-Vax group, we conclude the following:

- There was no significant difference in age (Figure 1a) or BMI.
- In the No-Vax group, a significantly lower prevalence of the male gender was observed (*p* = 0.0007).
- The percentage of actual and former smokers was significantly higher in the No-Vax group (*p* = 0.0003).
- The Charlson comorbidity index (Figure 1b) and each comorbidity were not significantly different, except for arterial hypertension that was significantly less prevalent in the No-Vax group (*p* = 0.0041).
- The number of days between the onset of symptoms and the hospital admission was significantly higher in the No-Vax group (*p* = 0.000), and the No-Vax survivors needed a significantly shorter hospital stay (*p* = 0.0021).
- Upon hospital admission, No-Vax patients showed a significant greater involvement of lung parenchyma, as assessed by the HRCT Chung score (*p* < 0.0001) and a worse PaO_2_/FiO_2_ ratio (*p* < 0.0001).
- Concerning the respiratory supports, in the No-Vax group there was a significantly more frequent use of HFNC (*p* < 0.0001) and a less frequent use of CPAP/NIV (*p* < 0.0001).
- The occurrence of pulmonary embolisms was significantly less frequent in the No-Vax group (*p* = 0.0049).
- A greater percentage of No-Vax patients died as compared with the Pre-Vax group, although the data did not reach statistical significance (*p* = 0.08).
- With respect to the laboratory data, AST, LDH, and NLR were significantly higher in No-Vax patients (*p* = 0.0225, *p* < 0.0001, *p* < 0.0001, respectively).

The identification of the variants of SARS-CoV-2 was available for 86% of No-Vax patients. When they were subdivided according to the SARS-CoV-2 variant, Delta or Omicron, and compared with the Pre-Vax group, each variant subgroup showed a significantly greater involvement of lung parenchyma, as assessed by the HRCT Chung score (*p* < 0.0001, *p* = 0.0009, respectively), and a worse PaO_2_/FiO_2_ ratio (both *p* < 0.0001), while there was no significant difference in the rates of deaths.

### 3.2. No-Vax vs. Vax Group

- A statistically higher number of patients was present in the No-Vax group in comparison with the Vax group (72.4% vs. 27.6%, *p* < 0.0001).
- Patients belonging to the Vax group were significantly older (*p* = 0.0097) (Figure 1a) and did not show a statistically different BMI.
- There was no significant difference in the prevalence of the male gender or in the percentage of actual and former smokers.
- Vax patients showed a significantly higher Charlson comorbidity index (*p* < 0.0001) (Figure 1b); in particular, arterial hypertension (*p* = 0.0371), COPD (*p* = 0.0142), and malignancies (*p* = 0.0477) were significantly more prevalent.
- Upon admission, Vax patients showed a significantly lower HRCT Chung score (*p* = 0.0015) (Figure 1c) and a PaO_2_/FiO_2_ ratio that was higher although not statistically significant (Figure 1d).
- During hospitalization, there were no statistically significant differences in the use of HFNC or CPAP/NIV.
- The occurrence of pulmonary embolism was not significantly different between the two groups.
- There was no significant difference in the percentages of patients who died between the two groups.
- With respect to the laboratory data, creatinine was significantly higher in Vax patients (*p* = 0.001), while AST and LDH were significantly higher in No-Vax patients (*p* = 0.0021, *p* = 0.0012, respectively). NLR was similar between the No-Vax and Vax groups.

The identification of the variants of the SARS-CoV-2 was available for 90% of the vaccinated patients, hospitalized during the most recent wave of the COVID-19 pandemic. The Delta variant was more prevalent in the No-Vax group (81.1% vs. 52.5%; *p* = 0.0013). On the contrary, the Omicron variant was relatively more frequent in the Vax group (32.5% vs. 16.2%, *p* = 0.0393).

In the Vax group, no significant differences were found between patients with the Omicron or Delta variants in terms of HRCT Chung score (10.0 [8.5–13.5] vs. 13.5 [11.3–15.0], *p* = 0.0979), PaO_2_/FiO_2_ ratio (98.0 [80.0–173.5] vs. 85.0 [69.5–127.0], *p* = 0.3666), or death rate (30.8% vs. 38.1%; *p* = 0.7271).

In the No-Vax group, there were no differences between the Omicron and Delta variants in terms of HRCT Chung score (15.0 [13.0–17.0] vs. 15.0 [13.5–17.0], *p* = 0.7437). However, a lower PaO_2_/FiO_2_ ratio was observed in patients with Omicron when compared to subjects infected with Delta (68.0 [58.0–82.5] vs. 83.0 [67.0–117.0], *p* = 0.0273), and the death rate was higher in patients affected by the Omicron variant with respect to Delta (70.6% vs. 41.1%; *p* = 0.0337).

## 4. Discussion

The main purpose of this study was to compare the clinical profile of unvaccinated patients with critical COVID-19 pneumonia hospitalized during the most recent COVID-19 pandemic with that of patients admitted to the same sub-intensive care unit before the start of the vaccination campaign.

According to the literature, advanced age and comorbidities, such as arterial hypertension, diabetes mellitus, and obesity, still represent the main risk factors for critical COVID-19 pneumonia [10]. However, in the group of No-Vax patients, the prevalence of the male gender was no longer observable, and there was a significantly higher percentage of actual or former smokers. In line with our results, recent studies have demonstrated that smoking patients hold more negative attitudes toward vaccines in general and are more likely to be undecided or unwilling to be vaccinated against SARS-CoV-2, compared with never and former smokers [17].

Regarding the clinical presentation of No-Vax patients, it appears to be more severe than that of the Pre-Vax group. In fact, at hospital admission, they show a greater extent of pneumonia on the chest CT scan and have a more severe respiratory failure. A higher percentage of deaths occurs in the No-Vax group, although the increase is not statistically significant. This worse clinical course occurs despite the greater knowledge of the disease and its treatment by the healthcare personnel, as shown by the significantly shorter hospital stay of the No-Vax survivors in comparison with the Pre-Vax. During the latest wave of the COVID-19 pandemic, there has been a different management of both acute respiratory failure and COVID-19 pneumonia. As shown by the results of the present study, there has been a greater use of HFNC, while CPAP/NIV are less frequently adopted. Being an aerosol-generating procedure, HFNC was avoided during the first COVID-19 waves due to concerns about the spread of infection. However, various studies have shown that the benefits of HFNC may outweigh their risks during COVID-19 pandemics. Although HFNC should be utilized in negative-pressure rooms, some studies have demonstrated that its use did not result in an increased aerosol formation with respect to conventional oxygen therapy [18,19]. Compared to CPAP/NIV, HFNC represents a non-invasive strategy, which requires less skill by the healthcare personnel and is thus associated with fewer complications. It allows the delivery of high oxygen concentrations, improved carbon dioxide removal, and increased mucociliary clearance [20]. In addition, HFNC can be applied to patients in a prone position, a validated strategy to promote lung recruitment [21]. Overall, it has been demonstrated that HFNC may reduce the need for intubation in COVID-19 patients and the length of stay in intensive care units of COVID-19 survivors [22]. Regarding the treatment of COVID-19 pneumonia, although systemic corticosteroids were already administered to our patients during the first wave of the COVID-19 pandemic, as described in our previous study, the administration of prophylactic doses of anticoagulants to COVID-19 patients requiring an ICU-level care during the last pandemic wave, as recommended by NIH guidelines, has significantly reduced the incidence of thromboembolic complications [13,14]. In accordance with our results, Kurahara Y et al. found that the overall disease severity in the fourth wave was higher than that in the first to third waves, while they did not observe any significant increase in mortality [23].

We can speculate that the worse course of the disease in the No-Vax group in comparison with the Pre-Vax could be attributed to the higher morbidity of the Delta and Omicron variants, as shown by our results. In addition, a longer stay at home prior the hospitalization could be another cause of the increased severity of COVID-19 pneumonia in the No-Vax patients. As largely demonstrated in previous studies, many No-Vax subjects deny or underestimate the severity of the disease, do not believe in science, express concerns about the short time in which anti-SARS-CoV-2 vaccines have been produced, or are afraid of their side effects and safety [24,25,26].

Regarding the laboratory data, the higher levels of AST and LDH in the No-Vax in comparison with both the Pre-Vax and Vax groups could be related to their more severe respiratory failure. Hypoxia in COVID-19 patients could lead to hepatocellular necrosis through the marked increase in reactive oxygen species and the following release of hepatotoxic pro-inflammatory factors [27]. Abnormal levels of LDH can also result from reduced oxygenation [28]. NLR was higher in the No-Vax than in the Pre-Vax group. A meta-analysis suggested that higher NLR values on admission are associated with a higher risk of both severity and mortality of COVID-19 patients [29]. NLR is a known marker of systemic inflammation that has been widely used to predict the outcomes of patients with cardio-vascular diseases or sepsis [30,31]. A high NLR indicates an imbalance in the inflammatory response, which results in increased neutrophil and decreased lymphocyte counts. Inflammatory factors related to viral infection, such as IL-6, IL-8, and granulocyte colony-stimulating factor, could stimulate neutrophil production [32]. In contrast, systemic inflammation accelerates lymphocyte apoptosis, depresses cellular immunity, and decreases CD4+ and increases CD8+ suppressor T-lymphocytes [33]. However, no NLR consensus cut-off value has been established, especially for COVID-19 patients. Several studies have used an NLR cut-off ranging from 3.3 to 5.9 to predict severity [34,35], and between 7.9 and 11.8 to predict mortality [36,37]. In our study, we found a median value of NLR higher than 6 in the Pre-Vax group which was significantly increased in the No-Vax group. On the basis of its ability to regulate immune-related pathways and cytokine expression, traditional Chinese medicine has been widely employed for the treatment of mild to severe COVID-19 cases in China [38,39].

The clinical profile of the vaccinated patients admitted to our sub-intensive unit care is substantially different in comparison with that of the No-Vax patients. In line with the results of previous studies, vaccinated patients are older, with a mean age over 70 years, and with more comorbidities, as demonstrated by a significantly higher Charlson comorbidity index [9,40,41]. Upon hospital admission, Vax patients showed less pulmonary involvement on a chest CT and had a lower severity of respiratory failure, although this was not statistically significant, while they displayed similar mortality rates, probably related to their older age and higher prevalence of comorbidities. A recent study showed that when fully vaccinated patients are well matched with unvaccinated patients using their propensity scores, their probability to die significantly decreases [42]. As demonstrated by Tatjana Schwarz et al., older age can reduce both humoral and cellular immune responses to the BNT162b2 vaccine (Pfizer-BioNTech) [43]. Furthermore, in the elderly, concomitant diseases and/or their treatment can compromise the immune responses to vaccines. In our group of vaccinated patients, COPD and malignancies were significantly more frequent than in the No-Vax patients. A recent study showed that COPD represents an independent risk factor for hospitalization, ICU stay, and mortality in COVID-19 patients [44]. It has been hypothesized that COVID-19 pneumonia and/or the associated pulmonary vascular thromboembolic events may aggravate the impairment of respiratory function already present in COPD patients. Furthermore, a recent study demonstrated an increased expression of the SARS-CoV-2 receptor in the airways and lungs of COPD patients, allowing a more rapid diffusion of the virus in the distal airways and alveolar spaces, facilitating the evolution of the upper respiratory tract infection into interstitial pneumonia [45,46]. Finally, a reduced innate immune response to viruses has been demonstrated in COPD patients [47,48]. The higher rate of malignancies in the vaccinated patients may further compromise the immune response to the virus as a consequence of several factors: as the underlying malignancy by itself, the cytotoxic chemotherapy, and/or the prior or concomitant immunosuppressive treatments. Follow-up studies have shown that COVID-19 patients with cancers exhibit a lower seroconversion rate to anti-SARS-CoV-2 vaccines [49].

Regarding the laboratory data, we found higher levels of creatinine in the Vax group in comparison with the No-Vax. In previous meta-analyses, elevated levels of creatinine detected in patients affected by severe COVID-19 without chronic kidney diseases suggested a possible acute injury caused by SARS-CoV-2 infection [50]. The binding between SARS-CoV-2 and ACE2 receptors might activate angiotensin II and induce cytokine production leading to a state of hyper-coagulopathy, microangiopathy, and renal hypoxia [51,52].

In agreement with the results of previous studies that have unequivocally demonstrated the effectiveness of vaccination programs against SARS-CoV-2 infection in drastically reducing the burden of hospital admissions and deaths related to COVID-19, we observed a significantly lower prevalence of vaccinated patients affected by critical COVID-19 pneumonia (27%) in comparison with the unvaccinated (73%) admitted to our sub-intensive unit care [53,54]. Similarly, McAlister FA et al. observed that 91% of patients hospitalized in Alberta during wave three of the COVID-19 pandemic were not fully vaccinated [55]. However, the risk of breakthrough cases of severe COVID-19 after vaccination still remains, particularly among groups at a higher risk of severe disease such as those with advanced age and comorbidities [40,56].

In the group of No-Vax patients, in accordance with the epidemiological data in Italy related to the period between November 2021 and February 2022, the Delta variant was more frequently detected in comparison with the Omicron. However, in contrast to the results of a previous study showing a substantially reduced overall severity of the Omicron variant compared to the Delta, in our group of unvaccinated patients, infection by the Omicron variant was associated with a greater severity of respiratory failure and a higher rate of death [57]. In the group of vaccinated patients, no significant differences were observed in the course of the disease between the Omicron and Delta strains. Demographic changes, comorbidities, vaccination status, and therapies may impair the interpretation of the results related to the impact of the different variants of the virus, and studies conducted on larger populations are strongly required.

## 5. Conclusions

The clinical profile of unvaccinated patients hospitalized during the last pandemic wave has changed in comparison with that of patients hospitalized before the start of the vaccination campaign; there is no longer a prevalence of the male gender, and there is a higher percentage of actual and former smokers. The clinical course of the disease is more severe, as shown by the greater extent of the pneumonia on chest HRCT and the higher degree of respiratory failure. More deaths occur, although the increase is not statistically significant. Anti-SARS-CoV-2 vaccines have drastically reduced the number of vaccinated patients admitted to a sub-intensive care unit for critical COVID-19 pneumonia. In comparison with No-Vax patients, vaccinated patients are older and have more comorbidities, especially COPD and malignancies, and although they show a lesser extent of pneumonia on chest HRCT at admission, they display similar mortality rates, probably due to their risk factors.

## Figures and Tables

**Table 1:** Anthropometric, clinical, and bio-humoral characteristics in the Pre-Vax, No-Vax, and Vax groups.

	Pre-Vax	No-Vax	Vax	*p*	*p*
	(n = 132)	(n = 105)	(n = 40)	Pre-Vax vs. No-Vax	No-Vax vs. Vax
Age, years, median [IQR]	62.9 ± 12.9	64.5 ± 13.5	71.5 ± 10.8	0.63	**0.0097**
Male sex, %	76.52	55.66	70.0	**0.0007**	0.1331
BMI, kg/m^2^, median [IQR]	28.0 [27.0–31.0]	27.7 [26.0–31.2]	26.0 [24.0–28.0]	0.5	0.0542
Smokers, %	26.0	51.6	40.0	**0.0003**	0.1184
CC index median [IQR]	2.0 [1.0–4.0]	3.0 [1.0–4.0]	4.0 [3.0–6.0]	>0.9999	**<0.0001**
Arterial hypertension, (%)	70.63	51.96	72.50	**0.0041**	**0.0371**
Obesity, (%)	37.50	29.29	17.50	0.2509	0.2001
Diabetes, (%)	25.21	23.53	37.50	0.8754	0.0999
COPD, (%)	13.91	12.87	32.50	0.8444	**0.0142**
Asthma, (%)	3.48	3.96	0.00	>0.9999	0.5747
Neoplasms, (%)	6.06	9.90	24.32	0.3261	**0.0477**
HRCT score median [IQR]	10.0 [7.0–13.0]	15.0 [13.5–17.0]	13.0 [10.0–15.0]	**<0.0001**	**0.0015**
PaO_2_/FiO_2_ median [IQR]	155.0 [110.0–233.0]	77.0 [63.5–106.0]	93.5 [71.3–162.0]	**<0.0001**	0.0518
HFNC, (%)	14.4	60.0	42.5	**<0.0001**	0.0644
CPAP/NIV, (%)	45.5	16.2	10.0	**<0.0001**	0.4352
Pulmonary embolism, (%)	27.73	12.38	5.0	**0.0049**	0.2375
Days in hospital, median [IQR]	23.0 [15.0–32.0]	16.0 [12.0–24.0]	18.5 [12.5–25.0]	**0.0021**	>0.9999
Death, (%)	33.85	45.71	35.0	0.08	0.26
CRP, mg/dL median [IQR]	9.4 [4.9–16.4]	8.9 [4.4–15.4]	9.3 [4.1–13.4]	>0.9999	>0.9999
IL-6, pg/mL median [IQR]	38.9 [21.0–79.3]	28.45 [18.8–64.6]	40.4 [15.2–84.6]	0.4307	>0.9999
D-dimer, ng/mL median [IQR]	389.0 [243.0–1104]	396.0 [237.8–1163]	464.0 [286.5–871.3]	>0.9999	>0.9999
PCT, ug/L median [IQR]	0.14 [0.07–0.36]	0.12 [0.07–0.35]	0.19 [0.08–0.45]	>0.9999	0.7100
AST, U/L median [IQR]	38.0 [25.0–59.0]	48.5 [34.0–73.3]	36.5 [19.0–47.0]	**0.0225**	**0.0021**
ALT, U/L median [IQR]	35.0 [21.0–70.0]	36.0 [27.5–71.0]	27.0 [19.0–43.8]	>0.9999	0.0522
LDH, IU/L median [IQR]	305.0 [240.0–483.0]	448.0 [343.5–649.8]	370.0 [245.3–473.3]	**<0.0001**	**0.0012**
Azotemia, mg/dL median [IQR]	50.0 [38.0–69.0]	56.0 [44.0–75.6]	79.0 [44.8–113.8]	0.2521	0.1048
Creatinine, mg/dL median [IQR]	0.8 [0.7–1.0]	0.8 [0.6–0.9]	1.0 [0.8–1.7]	0.4208	**0.0010**
Glycemia, mg/dL median [IQR]	122.0 [101.0–170.0]	125.5 [105.3–174.3]	129.5 [107.5–183.0]	>0.9999	>0.9999
WBC, ×10^9^/L median [IQR]	7.9 [5.86–11.3]	8.5 [5.5–11.5]	9.4 [6.0–12.4]	>0.9999	>0.9999
Lymphocytes,% median [IQR]	11.9 [6.9–16.3]	7.5 [5.0–11.2]	8.5 [6.0–12.8]	**<0.0001**	>0.9999
Neutrophils,% median [IQR]	80.7 [75.1–86.7]	86.8 [81.7–90.4]	84.7 [78.5–89.3]	**<0.0001**	0.7678
NLR median [IQR]	6.87 [4.59–12.84]	11.60 [7.36–17.94]	9.85 [6.42–14.88]	**<0.0001**	>0.9999
CRP, mg/dL median [IQR]	9.4 [4.9–16.4]	8.9 [4.4–15.4]	9.3 [4.1–13.4]	>0.9999	>0.9999

Abbreviations: BMI, body mass index; CCI, Charlson comorbidity index; HRCT, high-resolution computed tomography; PaO_2_, partial pressure of oxygen in the arterial blood; FiO_2_, fraction of inspired oxygen; HFNC, high-flow nasal cannula; CPAP, continuous positive airway pressure; NIV, non-invasive ventilation; CRP, C-reactive protein; IL-2R, interleukin-2 receptor; IL-6, interleukin-6; PCT, procalcitonin; AST, aspartate transaminase; ALT, alanine transaminase; LDH, lactate dehydrogenase; WBC, white blood cells; NLR, neutrophil/lymphocyte ratio.

## Data Availability

Data are available on request due to privacy restrictions.
